# Supplementary material for: A Cas-BCAR3 co-regulatory circuit controls lamellipodia dynamics
Source: eLife. 2021 Jun 25;10:e67078. doi: 10.7554/eLife.67078 (PMC8266394; doi:10.7554/eLife.67078)
Supplement: Source data 1. — Except where noted, blots were probed with anti-rabbit 800 and anti-mouse 700 and scanned on a Odyssey Infrared Imaging System. Individual files include lane designation and a brief explanation of antibodies used. Rb, rabbit. Ms, mouse. [file elife-67078-data1.zip › Figure Source Data Figure 1c and d.pdf]

Figure 1c

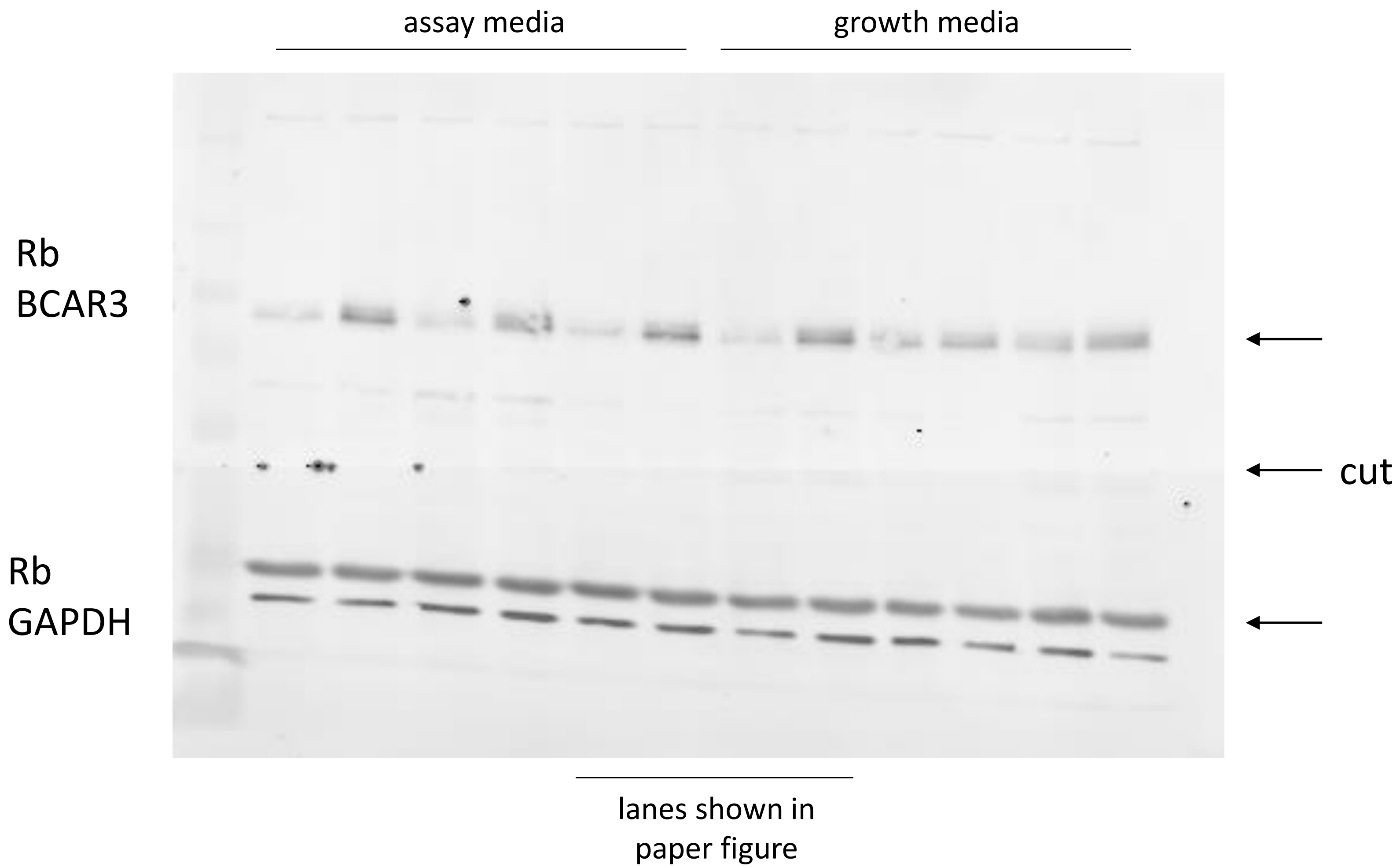

One blot was cut and probed separately for BCAR3 and GAPDH. Cropped out lanes are biological replicates

Figure 1d

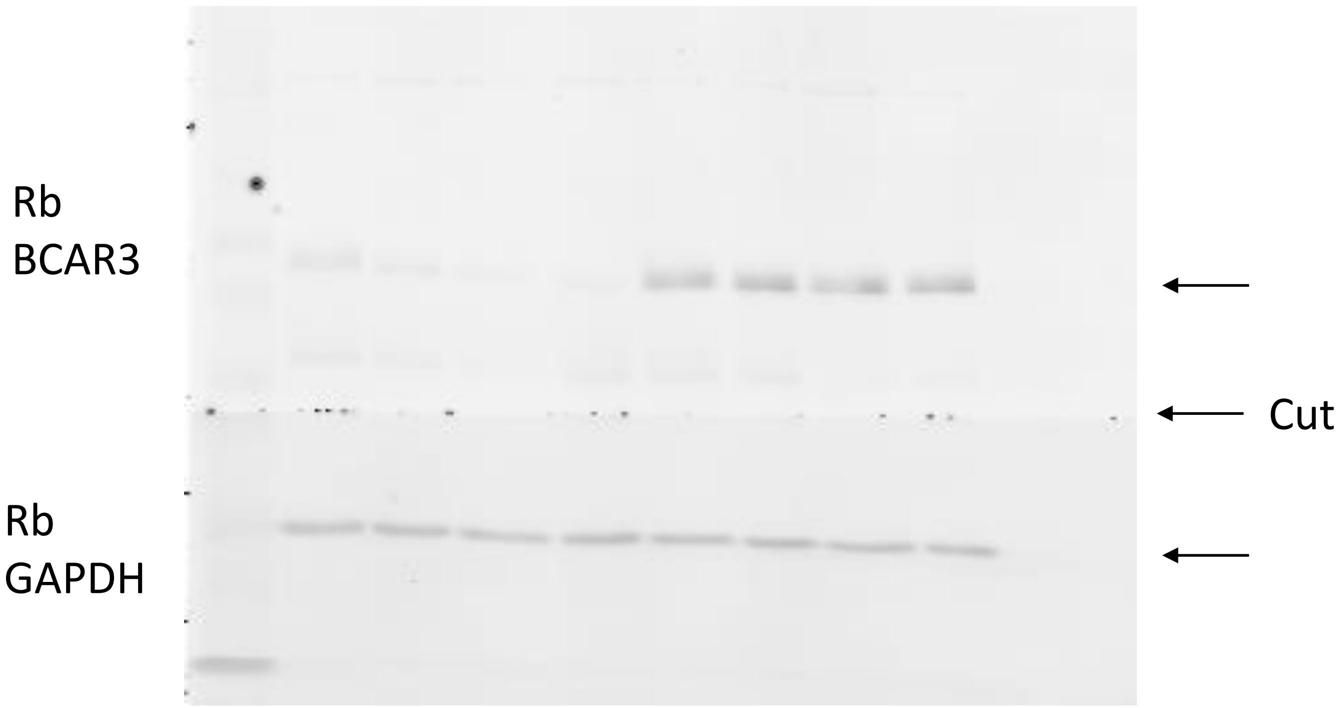

One blot was cut and probed separately for BCAR3 and GAPDH. Order of the lanes is the same as the figure.
