# Supplementary material for: A Cas-BCAR3 co-regulatory circuit controls lamellipodia dynamics
Source: eLife. 2021 Jun 25;10:e67078. doi: 10.7554/eLife.67078 (PMC8266394; doi:10.7554/eLife.67078)
Supplement: Source data 1. — Except where noted, blots were probed with anti-rabbit 800 and anti-mouse 700 and scanned on a Odyssey Infrared Imaging System. Individual files include lane designation and a brief explanation of antibodies used. Rb, rabbit. Ms, mouse. [file elife-67078-data1.zip › Figure Source Data Figure 2a.pdf]

Figure 2a

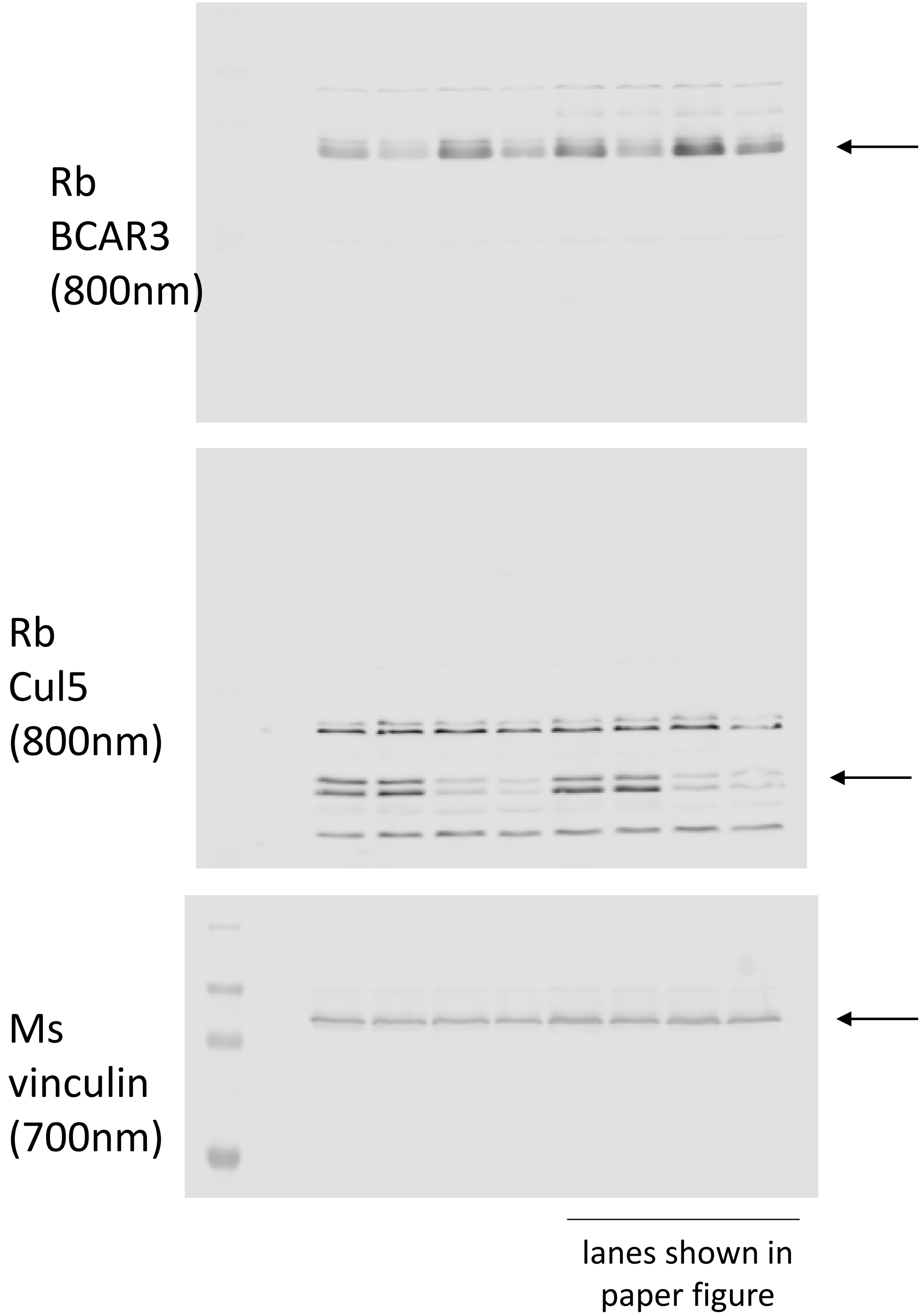

Order of the lanes is the same as the figure.
Two gels were run, one for Rb BCAR3 and Ms vinculin and one for Rb Cul5

Cropped out lanes were biological replicates
