# Supplementary material for: A Cas-BCAR3 co-regulatory circuit controls lamellipodia dynamics
Source: eLife. 2021 Jun 25;10:e67078. doi: 10.7554/eLife.67078 (PMC8266394; doi:10.7554/eLife.67078)
Supplement: Source data 1. — Except where noted, blots were probed with anti-rabbit 800 and anti-mouse 700 and scanned on a Odyssey Infrared Imaging System. Individual files include lane designation and a brief explanation of antibodies used. Rb, rabbit. Ms, mouse. [file elife-67078-data1.zip › Figure Source Data Figure 3a b c e f.pdf]

Figure 3a
(part)

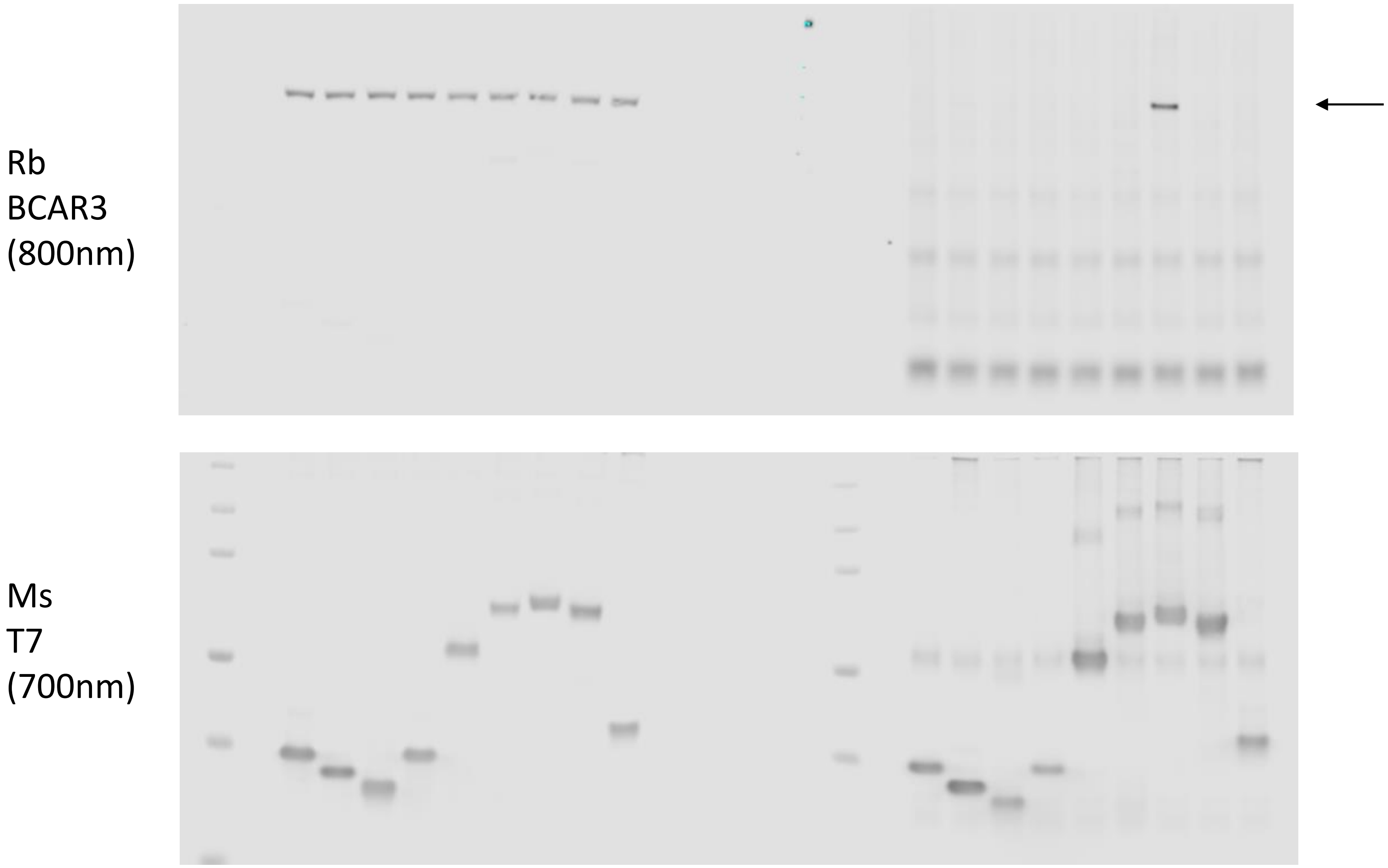

Two gels were run, blotted, probed and scanned at the same time. Lefthand blot shows lysates, righthand blot shows IPs. Each blot was probed first with Rb BCAR3 and Ms T7, followed by Rb Cas and Rb GAPDH. Order of the lanes is the same as the figure.

Figure 3a - continued

Rb
Cas
(800nm)

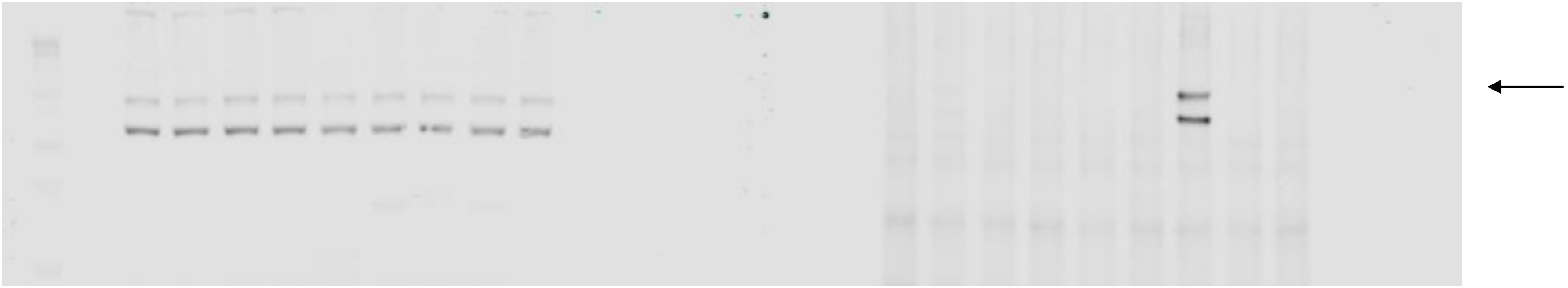

Rb
GAPDH
(800nm)

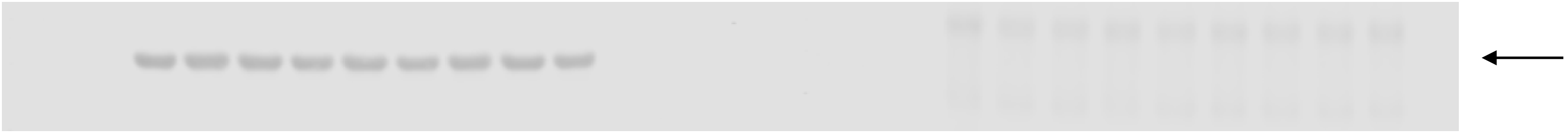

see previous legend...
The upper band in the Cas blot is Cas and the lower band is residual BCAR3

Figure 3b

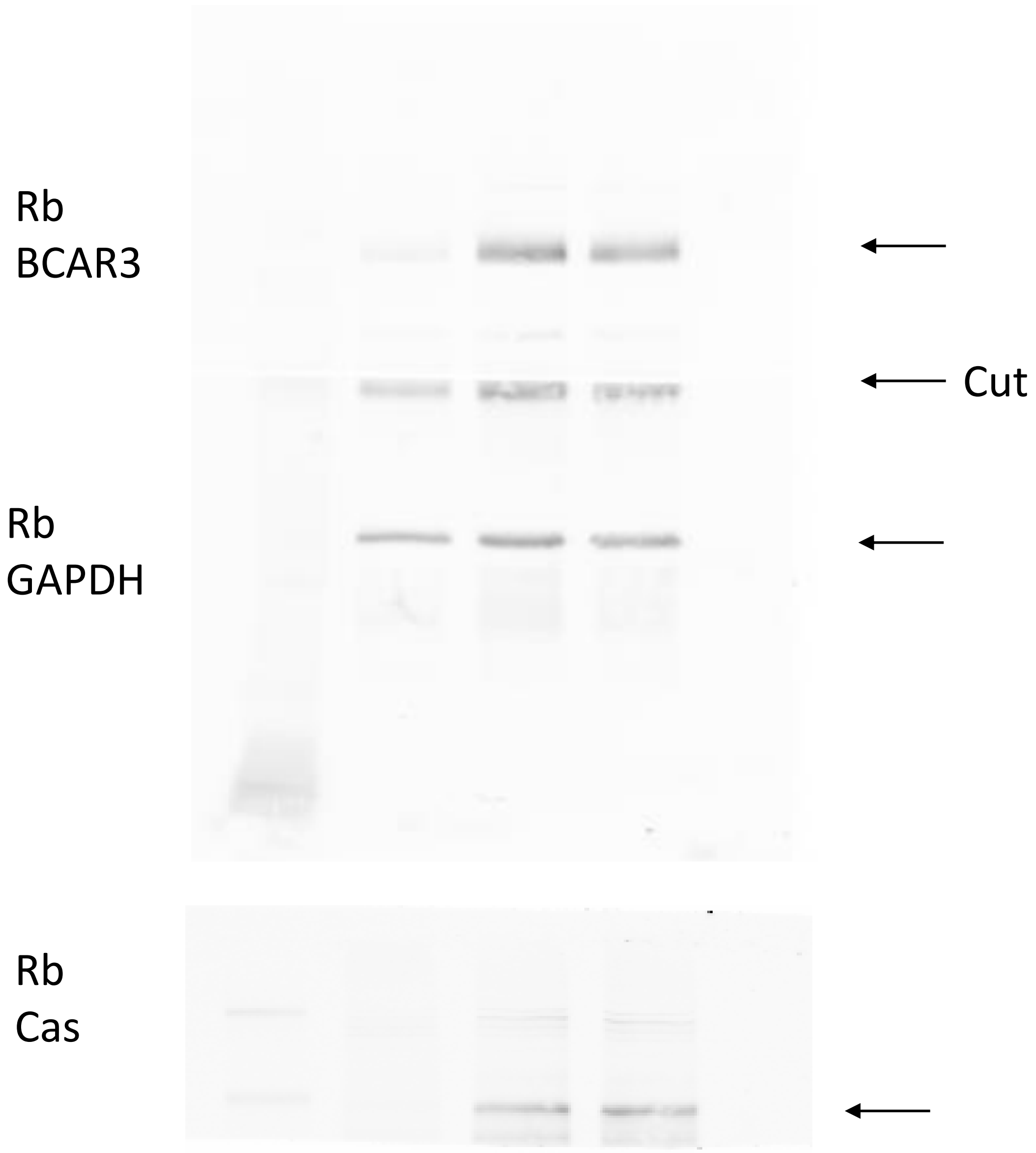

One blot was cut into two strips and probed for BCAR3 and GAPDH separately. The top strip was re-probed for Cas. In this experiment, the BCAR3 blot was cut above the BCAR3 band before probing with Cas antibody
Order of the lanes is the same as the figure.

Figure 3c

Rb
BCAR3
(800nm)

Ms
vinculin
(700nm)

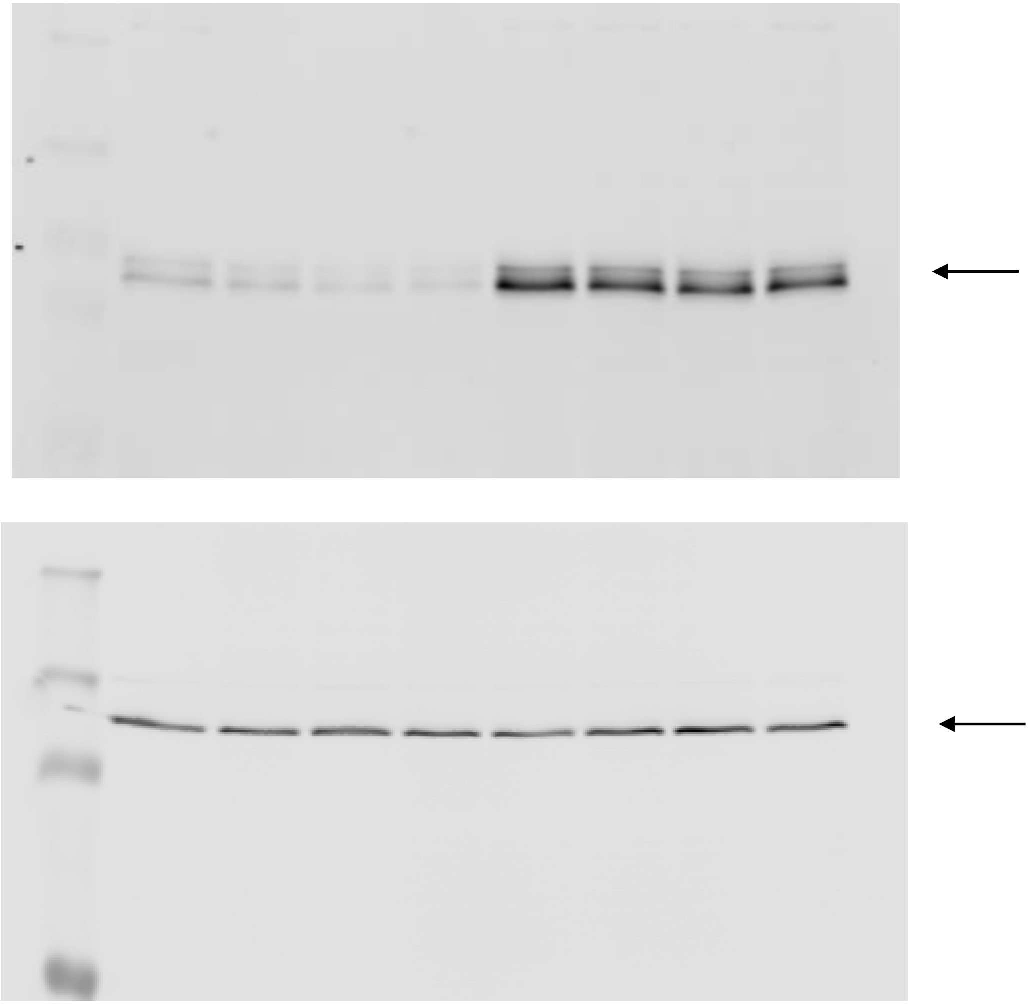

Order of the lanes is the same as the figure.

Figure 3e – left panel

Ms T7 (700nm)

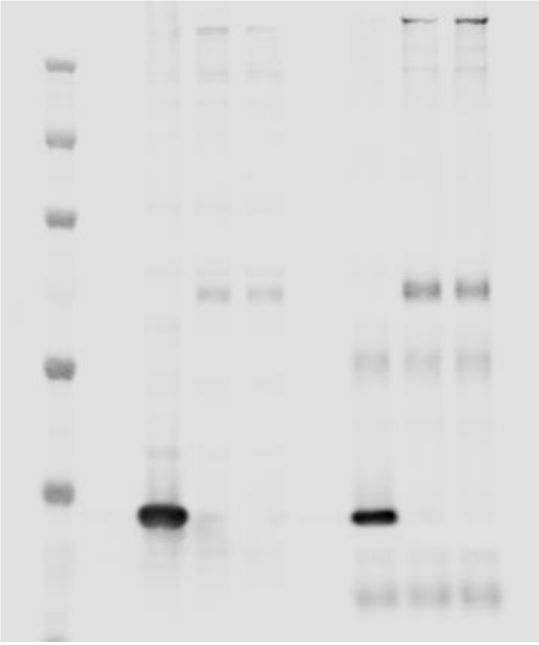

T7-SOCS6

T7-GFP

Rb V5 (800nm)

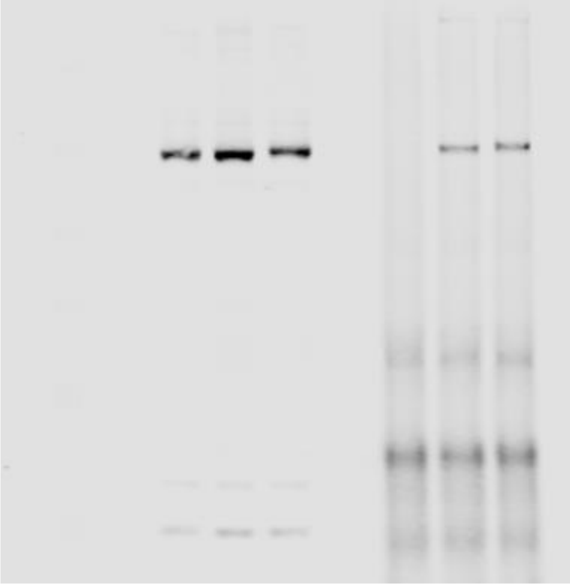

Rb GAPDH

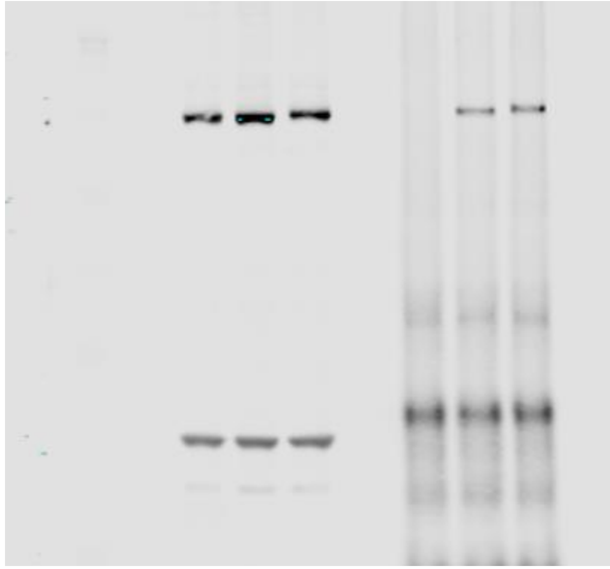

Blot was probed first with Ms T7 and Rb V5, followed by Rb GAPDH. Order of the lanes is the same as the figure.

Figure 3e – right panel

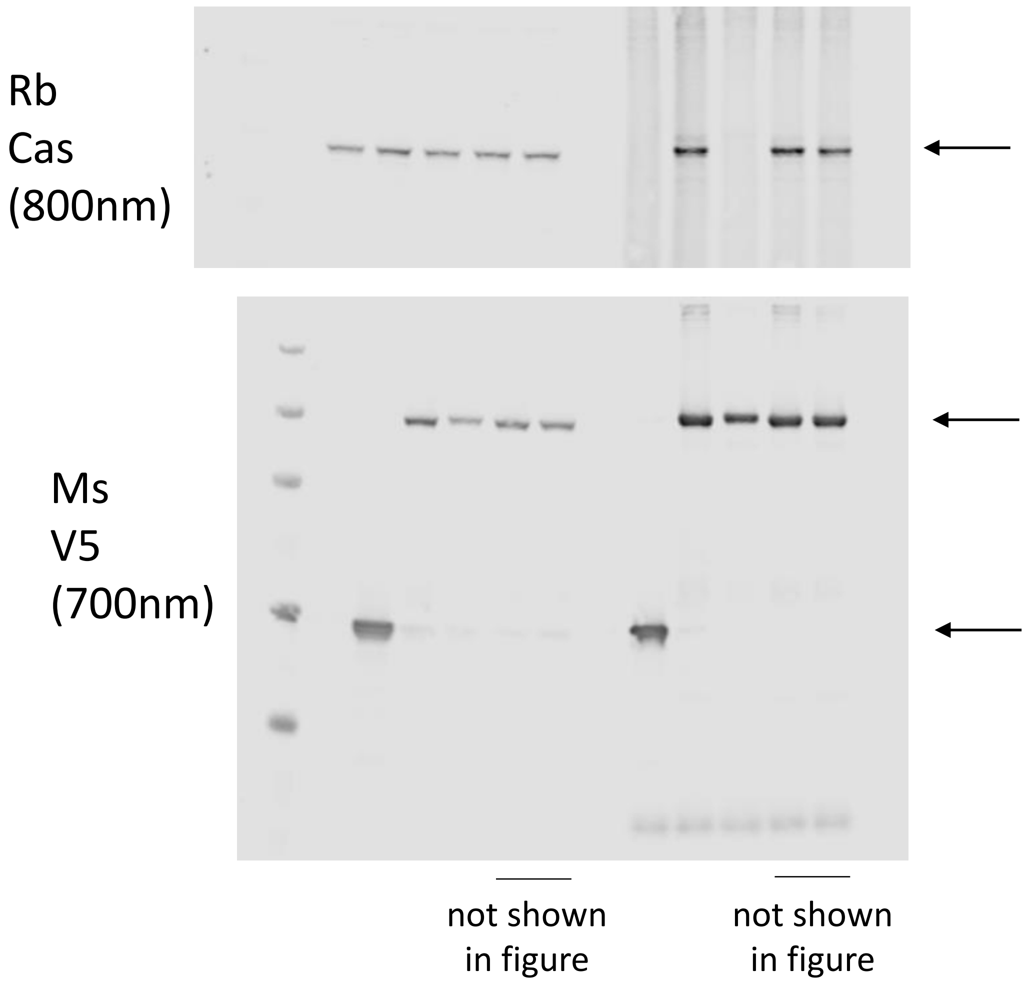

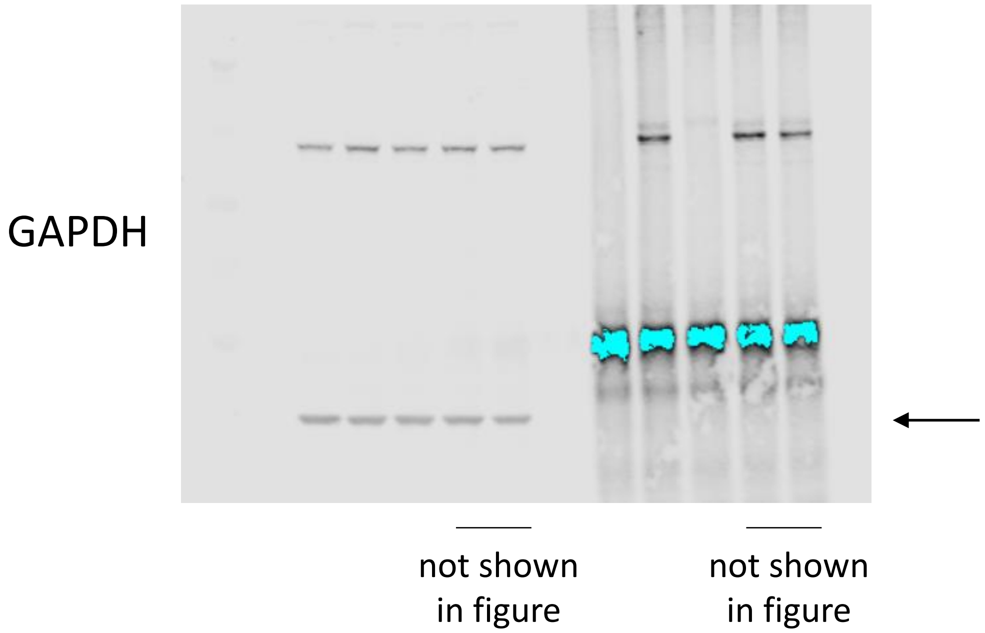

Blot was probed first with Rb Cas and Ms V5, followed by Rb GAPDH. The final two lanes of the lysate and IP show BCAR3 mutants that are not shown in this panel of the figure in the paper. Otherwise, the order of the lanes is the same as the figure.

Figure 3f

Ms
T7
(700nm)

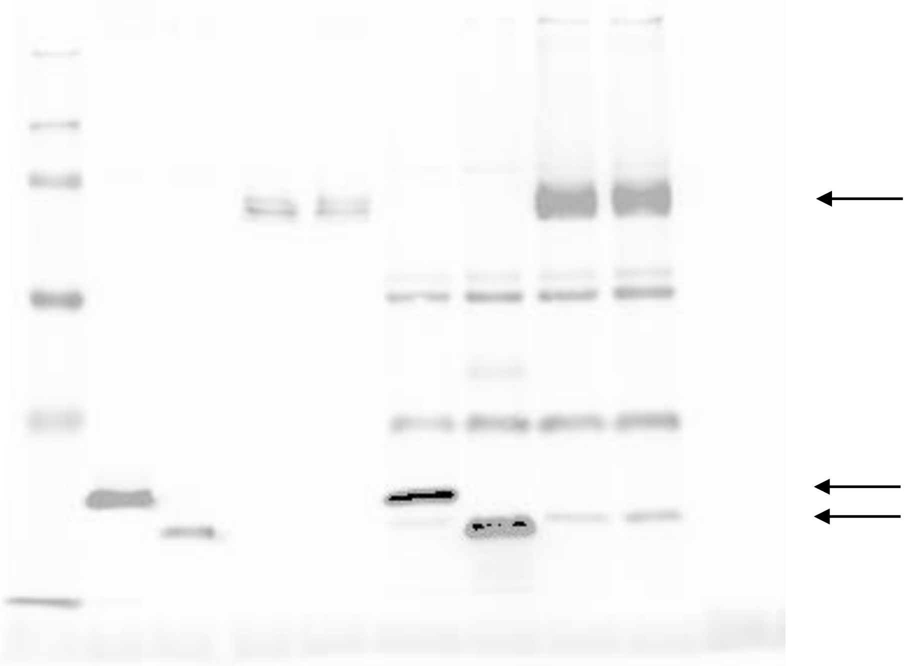

Rb
FLAG
(ECL)

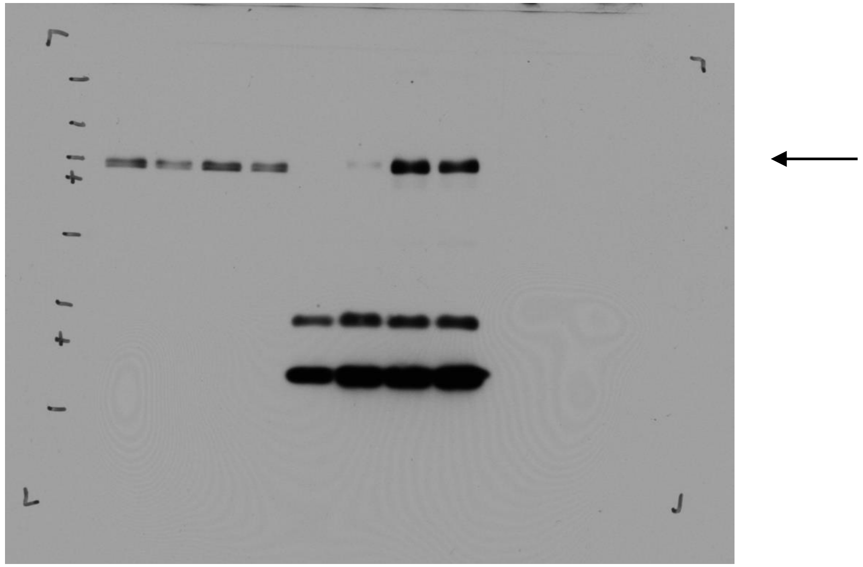

Rb
Cas
(800nm)

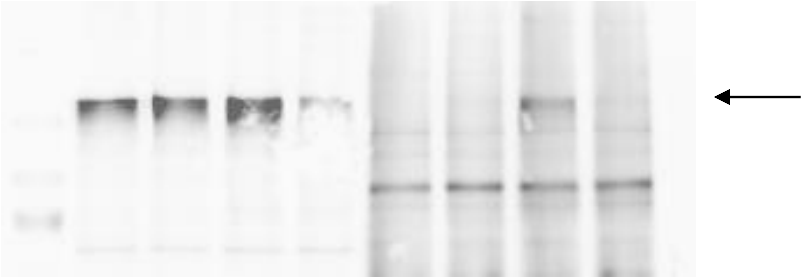

Rb
GAPDH

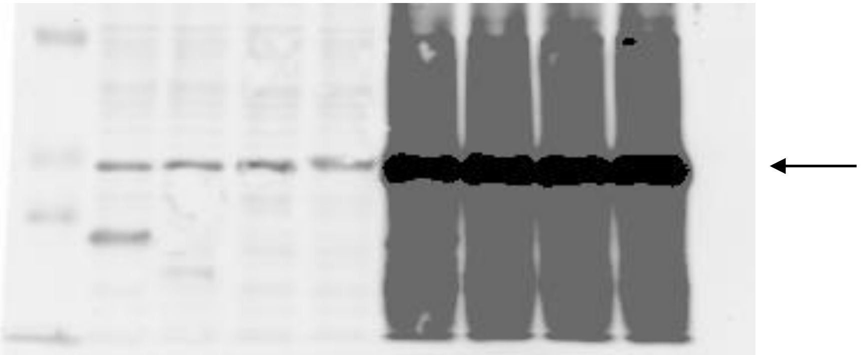

Order of the lanes is the same as the figure.
