# Supplementary material for: A Cas-BCAR3 co-regulatory circuit controls lamellipodia dynamics
Source: eLife. 2021 Jun 25;10:e67078. doi: 10.7554/eLife.67078 (PMC8266394; doi:10.7554/eLife.67078)
Supplement: Source data 1. — Except where noted, blots were probed with anti-rabbit 800 and anti-mouse 700 and scanned on a Odyssey Infrared Imaging System. Individual files include lane designation and a brief explanation of antibodies used. Rb, rabbit. Ms, mouse. [file elife-67078-data1.zip › Figure Source Data Figure 4a b d e.pdf]

Figure 4a

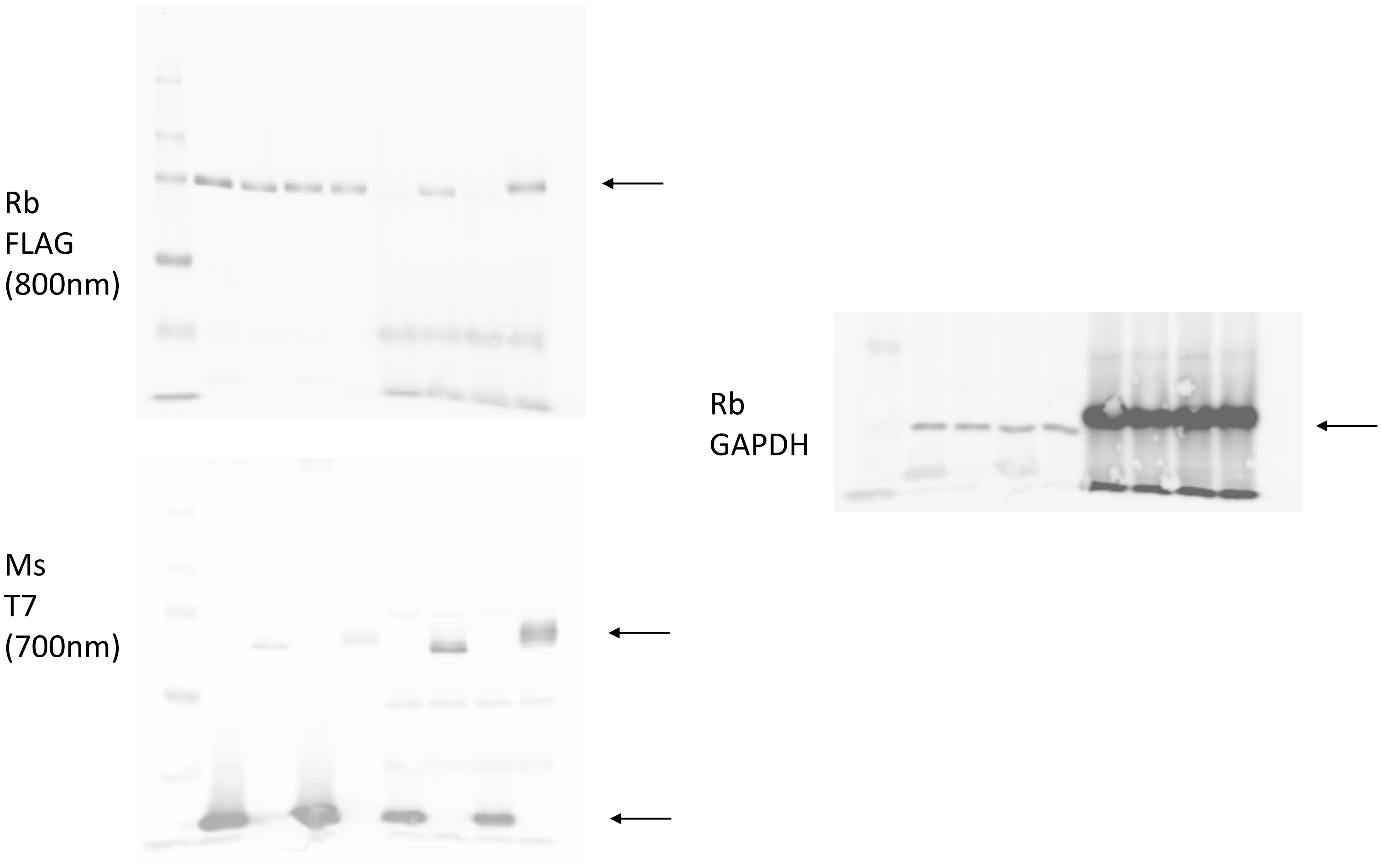

Order of the lanes is the same as the figure.

Figure 4b

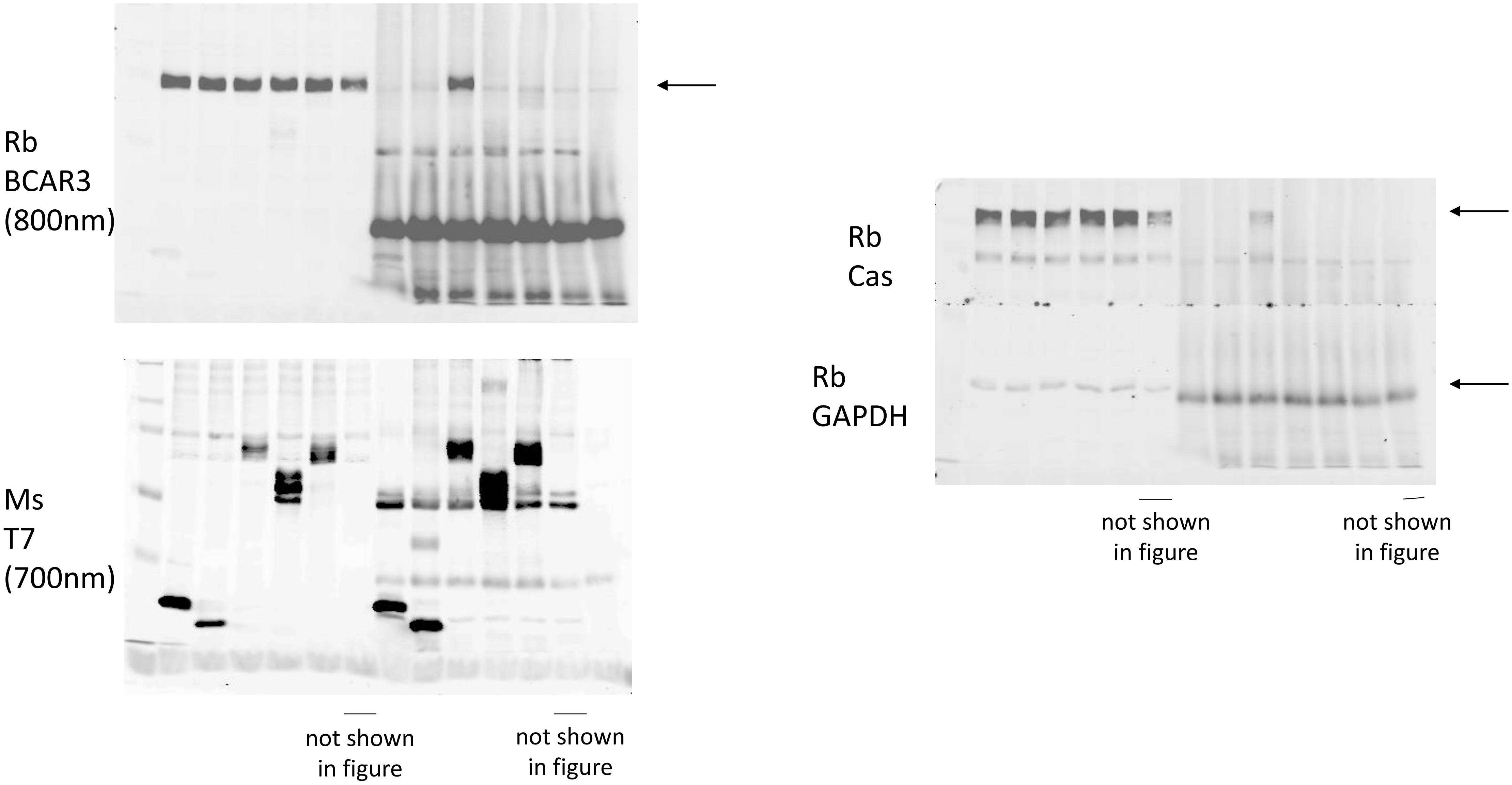

Blot was probed first with BCAR3 and T7, followed by Cas and GAPDH. The final lane of the lysate and IP show a SOCS6 mutant that is not shown in this figure in the paper. Otherwise, the order of the lanes is the same as the figure.

Figure 4d

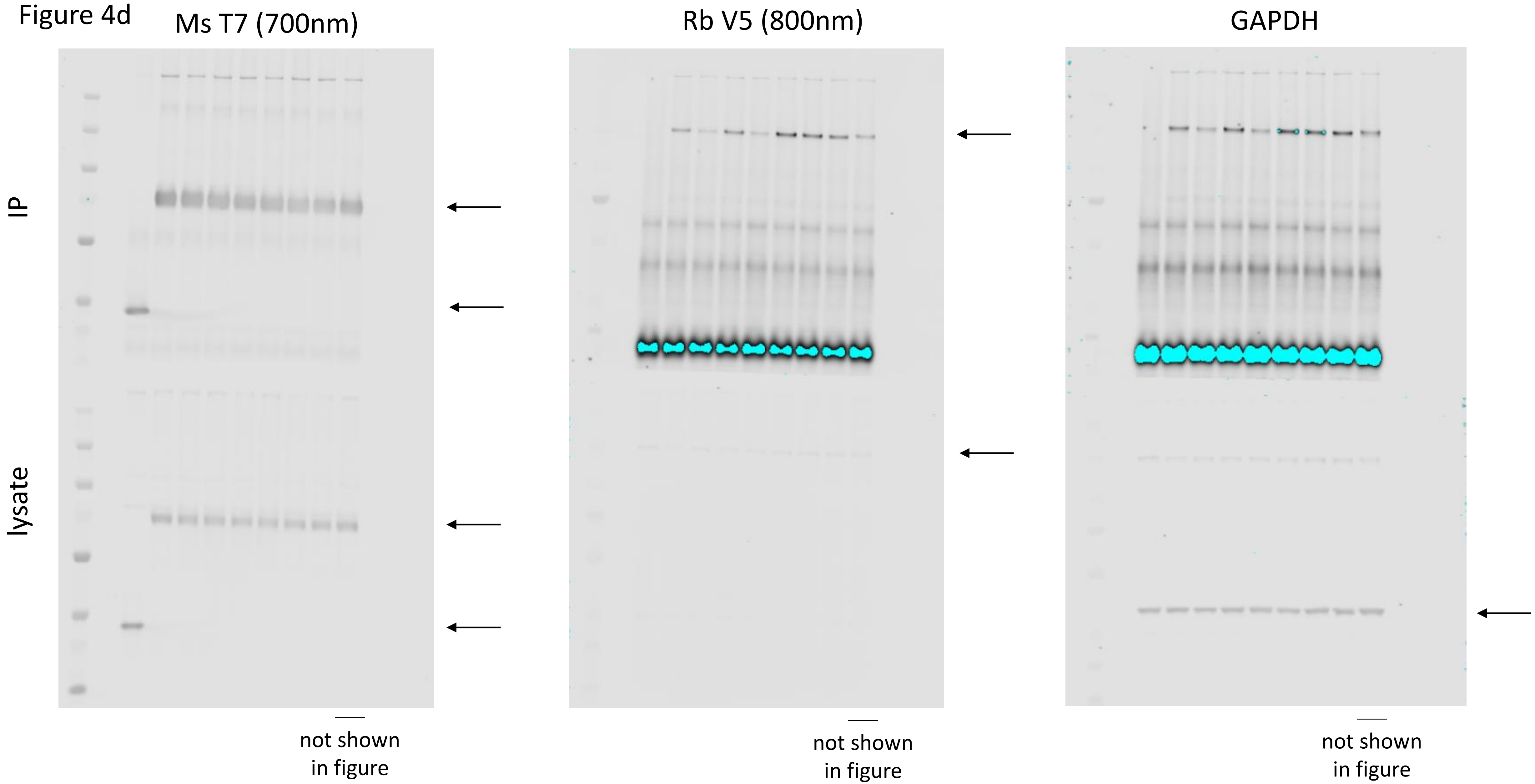

Two gels were run, one for IPs and one for lysates. Blot was probed first with T7 and V5, followed by GAPDH. The final lane of the lysate and IP show a BCAR3 mutant that is not shown in this figure in the paper. Otherwise, order of the lanes is the same as the figure.

Figure 4e

Ms
T7
(700nm)

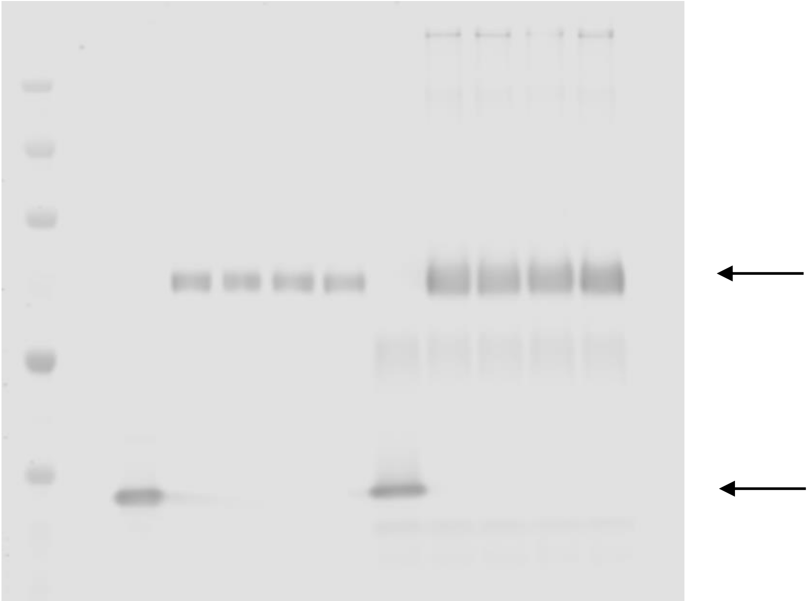

Rb
V5
(800nm)

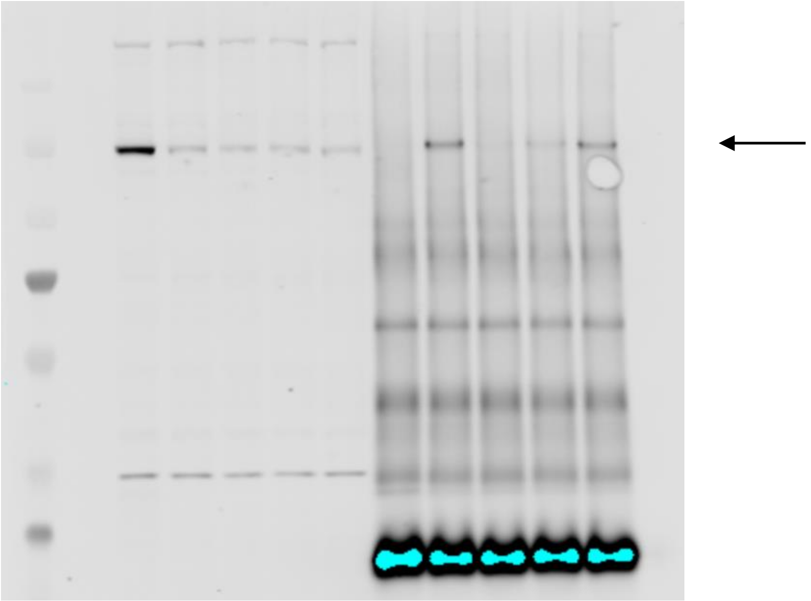

GAPDH

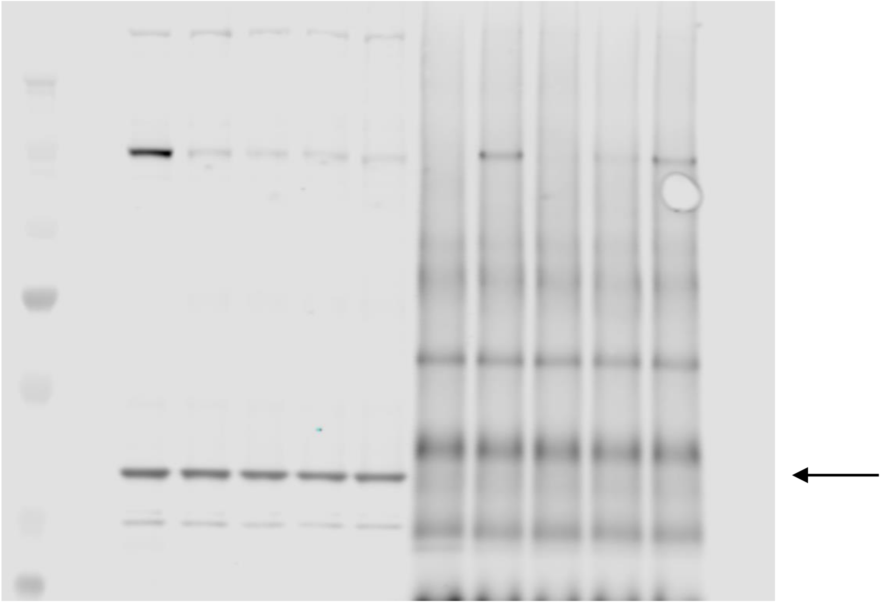

Blot was probed first with T7 and V5, followed by GAPDH. Order of the lanes is the same as the figure.
