# Supplementary material for: A Cas-BCAR3 co-regulatory circuit controls lamellipodia dynamics
Source: eLife. 2021 Jun 25;10:e67078. doi: 10.7554/eLife.67078 (PMC8266394; doi:10.7554/eLife.67078)
Supplement: Source data 1. — Except where noted, blots were probed with anti-rabbit 800 and anti-mouse 700 and scanned on a Odyssey Infrared Imaging System. Individual files include lane designation and a brief explanation of antibodies used. Rb, rabbit. Ms, mouse. [file elife-67078-data1.zip › Figure Source Data Figure 5a b c d.pdf]

Figure 5a

Rb
BCAR3
(800nm)

tagged
endogenous

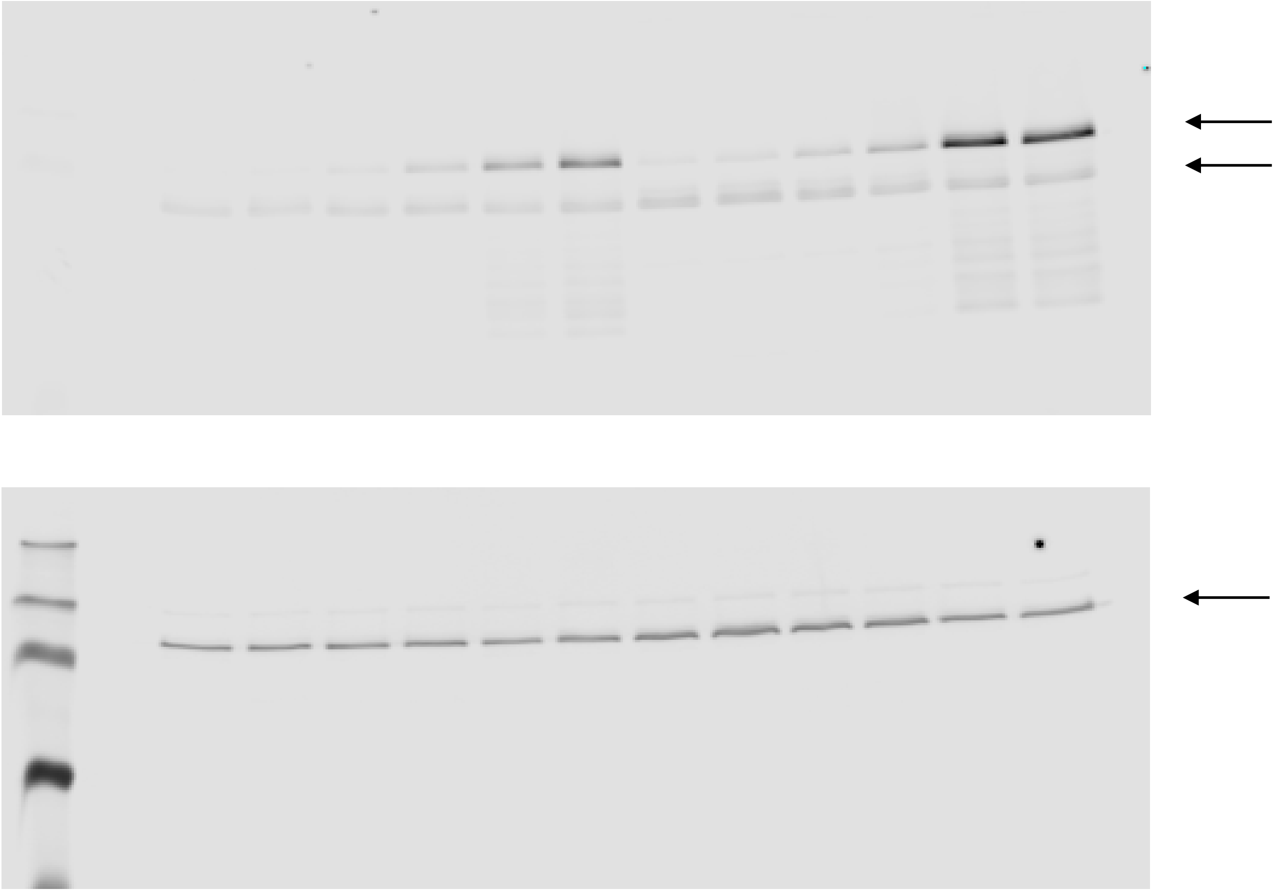

Ms
vinculin
(700nm)

Order of the lanes is the same as the figure.

Figure 5b

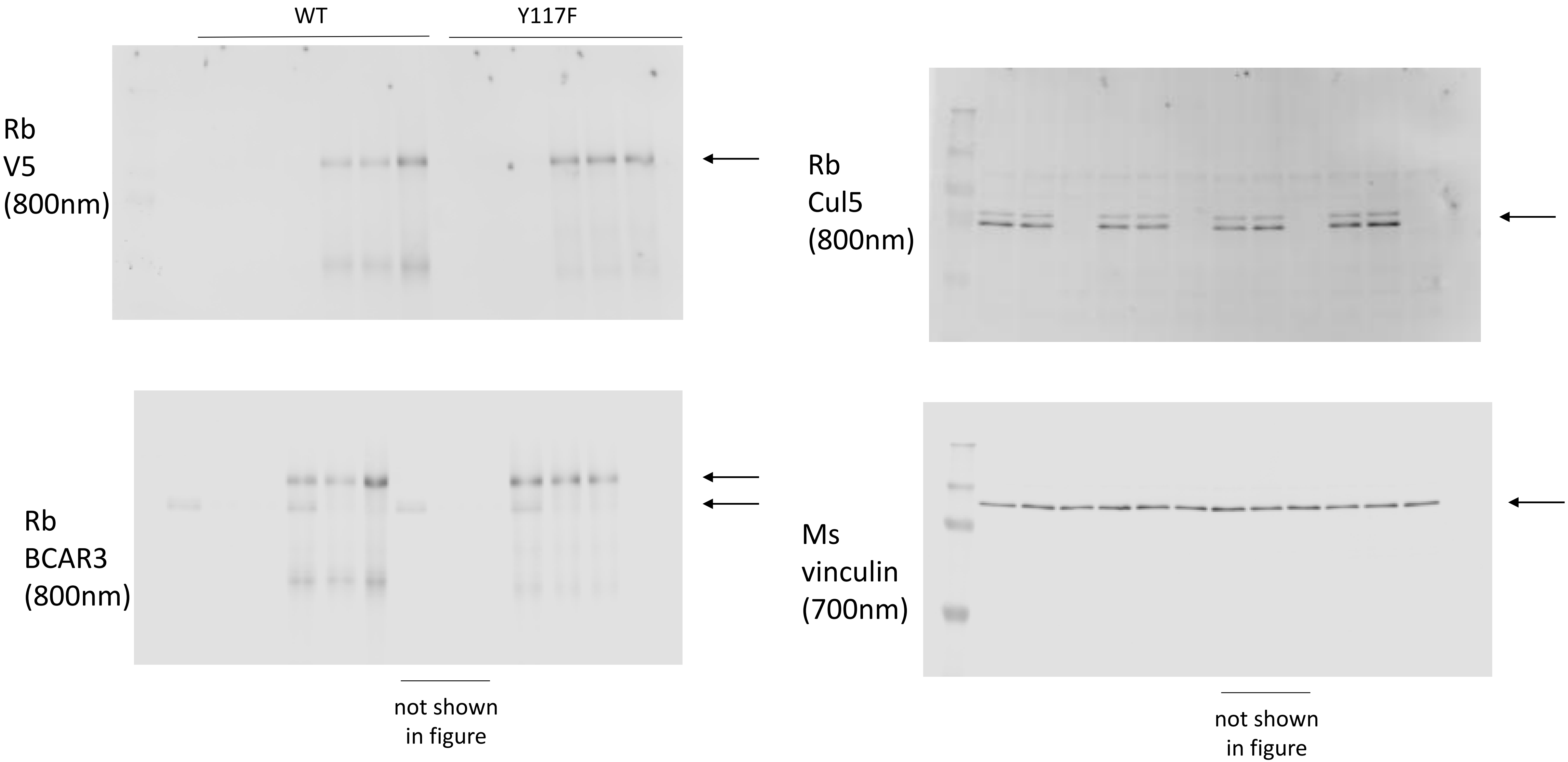

Three gels were run. One was probed with RbBCAR3, one with Rb V5 (Ms V5 antibody was back-ordered) and one with Rb Cul5 and Ms vinculin. BCAR3 Y117F conditions without dox were removed due to redundancy. Otherwise, order of the lanes is the same as the figure.

Figure 5c

Rb
BCAR3
(800nm)

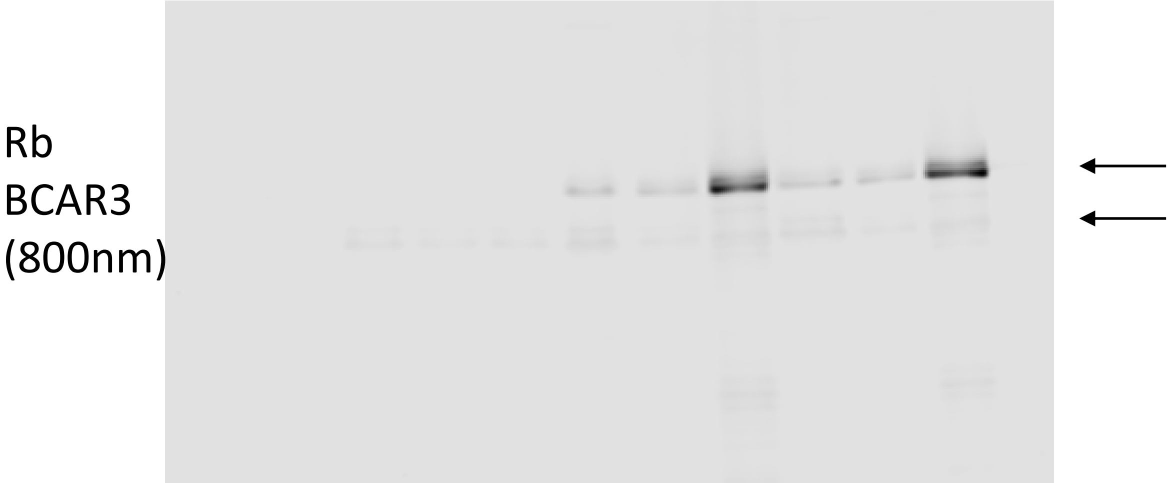

Rb
Cul5
(800nm)

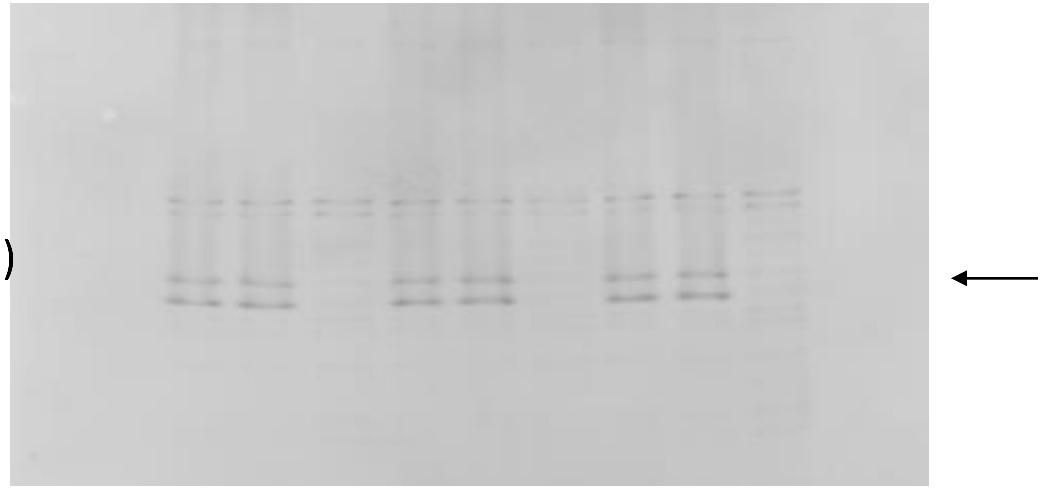

Ms
V5
(700nm)

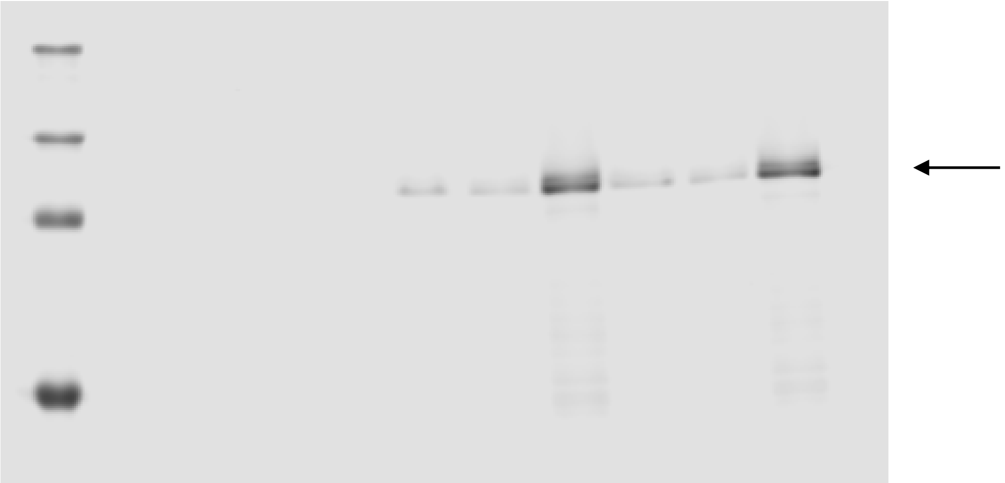

Ms
vinculin
(700nm)

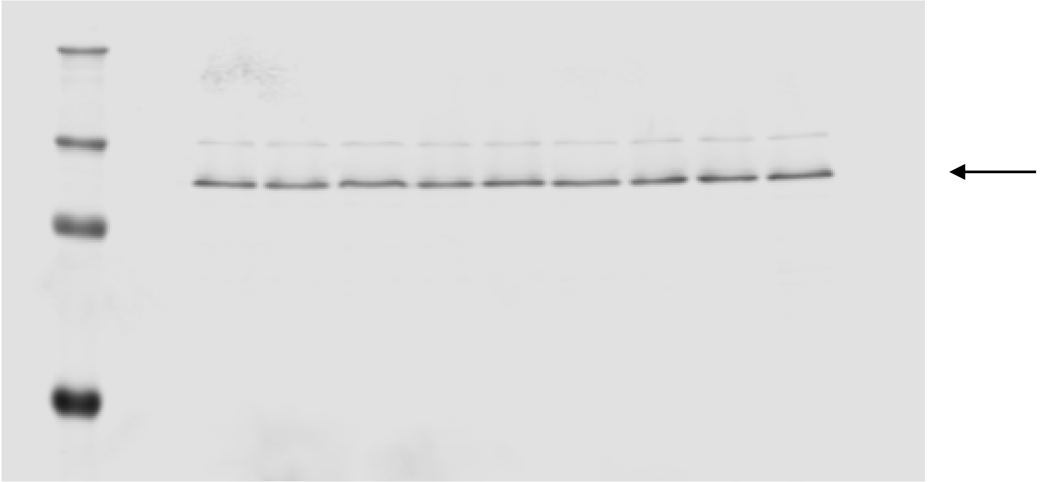

Two blots were run. One was probed with Rb BCAR3 and Ms V5, and one with Ms vinculin and Rb Cul5. Order of the lanes is the same as the figure.

Figure 5d – BCAR3 WT and R177K panels

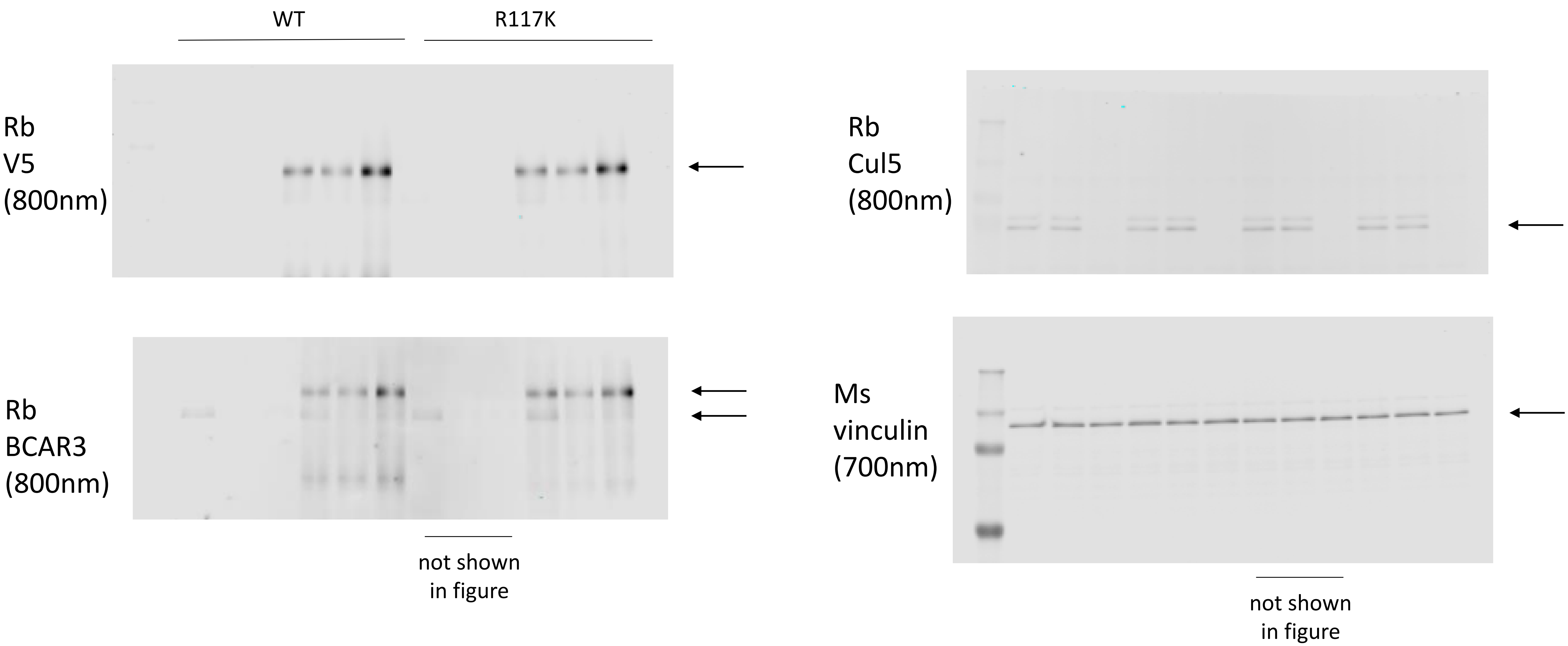

Three gels were run. One was probed with RbBCAR3, one with Rb V5 and one with Rb Cul5 and Ms vinculin.
BCAR3 R177K conditions without dox were removed due to redundancy. Otherwise, order of the lanes is the same as the figure.

Figure 5d – BCAR3 EE panels

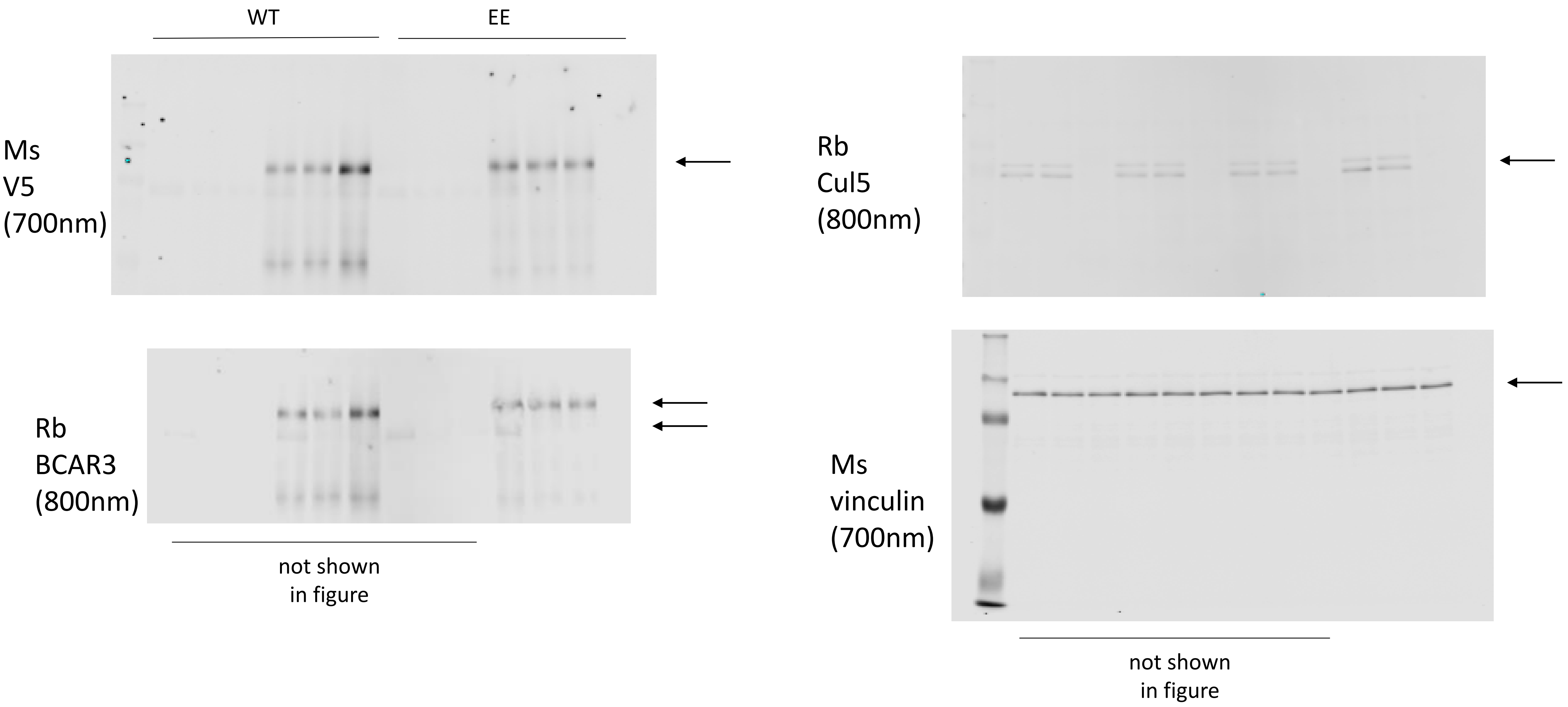

Three gels were run. These gels were run at the same time as Figure 5d R177K panels. One was probed with RbBCAR3, one with Rb V5 and one with Rb Cul5 and Ms vinculin. All BCAR3 WT lanes and BCAR3 EE conditions without dox were removed due to redundancy. Otherwise, order of the lanes is the same as the figure.
