# Supplementary material for: A Cas-BCAR3 co-regulatory circuit controls lamellipodia dynamics
Source: eLife. 2021 Jun 25;10:e67078. doi: 10.7554/eLife.67078 (PMC8266394; doi:10.7554/eLife.67078)
Supplement: Source data 1. — Except where noted, blots were probed with anti-rabbit 800 and anti-mouse 700 and scanned on a Odyssey Infrared Imaging System. Individual files include lane designation and a brief explanation of antibodies used. Rb, rabbit. Ms, mouse. [file elife-67078-data1.zip › Figure Source Data Figure 7a and b .pdf]

Figure 7a

Rb
BCAR3
(800nm)

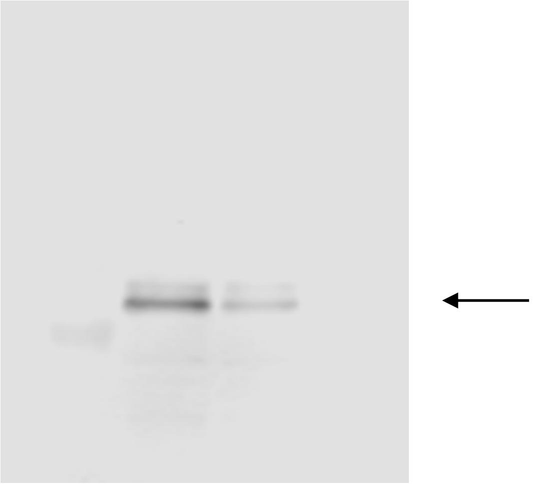

Rb
Cas
(800nm)

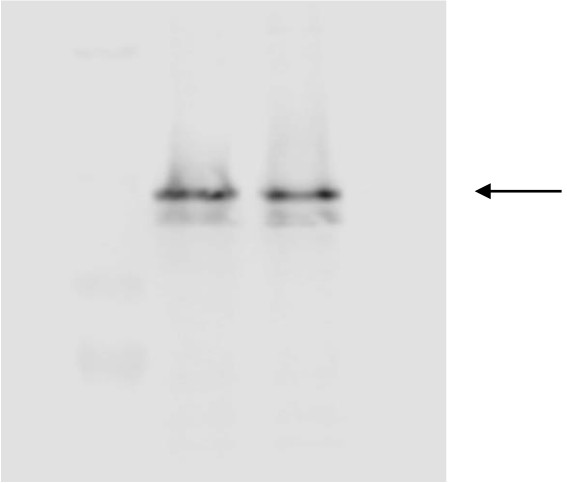

Ms
vinculin
(700nm)

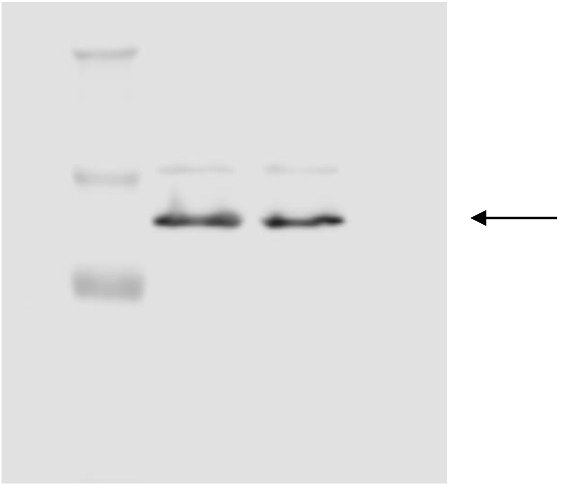

Duplicate lanes were run on the same gel and the blot cut to separate lanes that were probed with Rb BCAR3 or with Rb Cas and Ms vinculin. Order of the lanes is the same as the figure.

Figure 7b

Rb
Cas
(800nm)

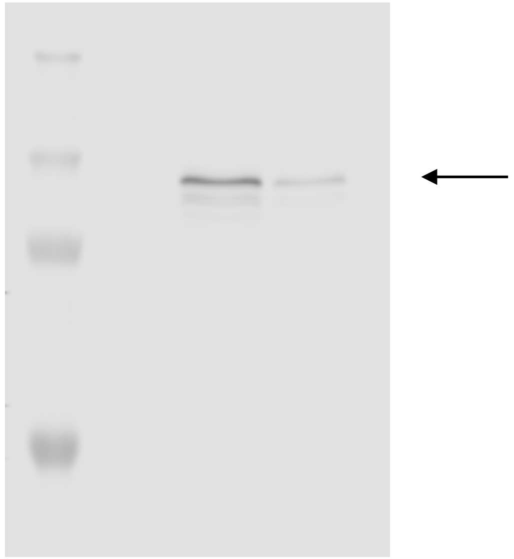

Ms
V5
(700nm)

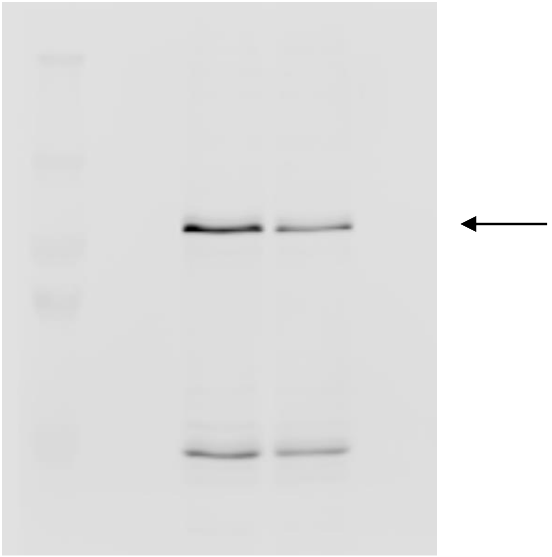

Ms
vinculin
(700nm)

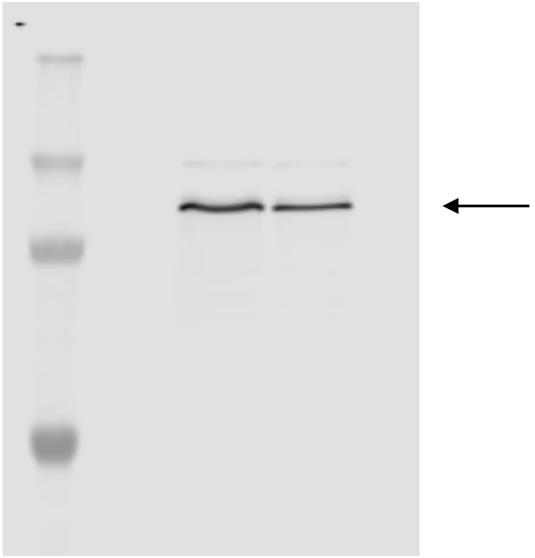

Duplicate lanes were run on the same gel and the blot cut to separate lanes that were probed with Rb Cas and Ms vinculin or with Ms V5.
Order of the lanes is the same as the figure.
