# Supplementary material for: A Cas-BCAR3 co-regulatory circuit controls lamellipodia dynamics
Source: eLife. 2021 Jun 25;10:e67078. doi: 10.7554/eLife.67078 (PMC8266394; doi:10.7554/eLife.67078)
Supplement: Source data 1. — Except where noted, blots were probed with anti-rabbit 800 and anti-mouse 700 and scanned on a Odyssey Infrared Imaging System. Individual files include lane designation and a brief explanation of antibodies used. Rb, rabbit. Ms, mouse. [file elife-67078-data1.zip › Figure Source Data Figure 8 supplement 3c .pdf]

Figure 8 – figure supplement 3

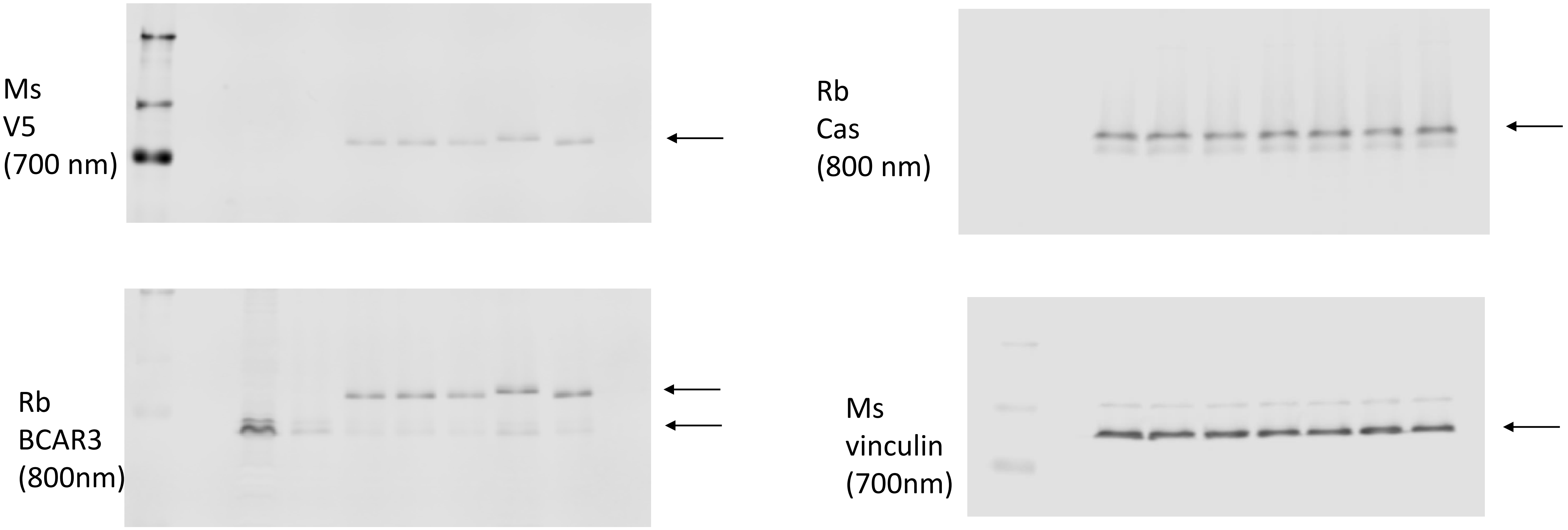

Duplicate gels were run on the same samples. One was probed with Rb BCAR3 and Ms V5 and one with Rb Cas and Ms vinculin. Order of the lanes is the same as the figure.
